# Supplementary material for: Evolution of a Cytoplasmic Determinant: Evidence for the Biochemical Basis of Functional Evolution of the Novel Germ Line Regulator Oskar
Source: Mol Biol Evol. 2021 Sep 22;38(12):5491–513. doi: 10.1093/molbev/msab284 (PMC8662646; doi:10.1093/molbev/msab284)
Supplement: msab284_Supplementary_Data [file msab284_supplementary_data.pdf]

Supplementary Materials

Evolution of a cytoplasmic determinant: evidence for the biochemical basis of functional evolution of the novel germ line regulator Oskar

Leo Blondel, Savandara Besse, Emily Rivard, Guillem Ylla, and Cassandra G. Extavour

These Supplementary Materials contain the following:

- Supplementary Text
- Supplementary Methods
- Supplementary References
- Legends for Supplementary Figures S1 through S12 (this document)
- Supplementary Figures S1 through S12 (this document)
- Legends for Supplementary Tables S1 through S5 (this document)
- Supplementary Tables S1 and S2 (this document)
- Supplementary Table S3 through S5 are provided in the GitHub repository for this study at https://github.com/extavourlab/Oskar_Evolution

Supplementary Text

oskar *expression levels in tissue-specific transcriptomes from a mosquito*

We examined all the TSA transcriptomes included in our original analysis, to determine which of them had a bioproject containing SRA sequences for more than one tissue type processed in the same manner, thus useful to derive relative *oskar* expression levels in distinct tissue types. Only one species, the mosquito *Aedes aegypti* (Diptera), satisfied this criterion. We examined the transcript quantification results provided by the authors of the original bioproject (Matthews, et al. 2016; their Supplementary Table 5). We also generated our own quantification analysis using the tool kallisto (Bray, et al. 2016)), with the TSA transcriptome (GFNA01) as an index, and all the SRA reads from the reported bioproject. We found that both quantification analyses yielded similar results (Supplementary Figure S5). Specifically, *oskar* transcripts were detectable in the brain, the ovaries, and what Matthews and colleagues (2016) describe as the "abdominal tip" of female mosquitoes (Supplementary Figure S5A, C). The latter tissue type is defined as the three posterior abdominal segments, which include the external and internal genitalia and ovipositor, but not the ovaries (2016). Expression in the abdominal tip was detected only in blood-fed female mosquitoes 96 hours after feeding, but not in non-blood-fed females (Supplementary Figure S5B, D). Given that female mosquito eggs mature after being blood-fed (Laurence 1977), we hypothesize that the abdominal tip *oskar* expression comes from matured eggs that have moved from the ovary into the bursa within the abdominal tip. In addition, in this dataset, expression levels of *oskar* are higher in the ovaries than in the brain (Supplementary Figure S5A, C). This could reflect a difference in transcriptional activity between the two tissue types, or it could reflect the possibility that the number of cells expressing *oskar* is lower in the brain than in the ovaries. *In situ* hybridization or a similar spatial expression approach will be needed to better understand the specific nature and number of cells that express *oskar* in these two mosquito organ systems.

oskar *expression levels in organ system-specific transcriptomes from a cricket*

We assessed the levels of *oskar* transcript in a methodologically comparable dataset of transcriptomes of 13 reproductive and nervous system tissues from males and females of the cricket *Gryllus bimaculatus* (Orthoptera) recently generated in our laboratory (Whittle, Kulkarni, Chung, et al. 2021; Whittle, Kulkarni, et al. 2021a). We detected the highest *oskar* expression levels in the female ovaries (mean value 114.10 transcripts per million (TPM)), followed by male testes and mixed-sex embryos (mean values 20.2 and 20.18 TPM respectively) (Supplementary Figure S6). Very low levels of *oskar* (mean 1.57 to 5.60 TPM) were detected in all other analyzed tissues (Supplementary Figure S6). The Table below shows the transcripts per million (TPMs) of *oskar* detected in each RNA-seq library of *G. bimaculatus* tissues obtained by Whittle, Kulkarni, et al. (2021b). For each tissue, two or three biological replicates were sequenced.

Sample	Replicate #	TPM
embryos	1	13.10
embryos	2	27.26
female_accessory gland	1	2.00
female_accessory gland	2	5.49
female_brain	1	3.74
female_brain	2	3.89
female_carcass	1	5.82

female_carcass	2	7.27
female_carcass	3	0.00
female_ovary	1	132.49
female_ovary	2	92.23
female_ovary	3	117.59
female_somatic_gonad	1	5.41
female_somatic_gonad	2	4.23
female_somatic_gonad	3	7.16
female_ventral_cord	1	3.16
female_ventral_cord	2	0.68
female_ventral_cord	3	5.83
male_accessory_gland	1	1.49
male_accessory_gland	2	1.73
male_brain	1	3.82
male_brain	2	1.63
male_carcass	1	3.74
male_carcass	2	0.00
male_somatic_gonad	1	2.55
male_somatic_gonad	2	7.81
male_testes	1	26.31
male_testes	2	14.10
male_ventral_cord	1	1.15
male_ventral_cord	2	1.98

Semi-quantitative RT-PCR assessment of **oskar** ***expression levels in a fly, a weevil, and a stick insect***

We used semi-quantitative RT-PCR to assess relative tissue-level *oskar* expression levels in male and female gonads and heads in three insect species: the fruit fly *Drosophila melanogaster* (Diptera; wild type strain Oregon R), the weevil *Callosobruchus maculatus* (Coleoptera), and the stick insect *Aretaon asperrimus* (Phasmatodea). In *D. melanogaster*, strong *oskar* expression was detected in female ovaries but none was detected in female heads (Supplementary Figure S7A). Barely detectable expression was also evident in male gonads and heads (Supplementary Figure S7A). This observation is consistent with transcriptome based-reports from FlyAtlas 2 (Leader, et al. 2017), but to our knowledge, no roles for oskar have been reported in these tissues in this fly.

In *C. maculatus*, expression of oskar was detected in male and female gonads and heads, as well as in embryos (Supplementary Figure 7B). Expression levels were highest in ovaries, followed by heads of both sexes, and lowest but still easily detectable in male gonads (Supplementary Figure S7B). The function of *oskar* in this weevil remains unknown, but these expression data suggest that it could function in one or both of the brain or gonads in both sexes.

In *A. asperrimus*, where we examined only female specimens, expression levels were higher in ovaries than in heads (Supplementary Figure S7C). As for the weevil, functional roles of *oskar* remain untested in this stick insect, but these expression data suggest that it may function in the female nervous and/or reproductive systems.

Supplementary Methods

Analysis of Aedes aegypti oskar transcript levels

To quantify the expression level of *oskar* in published transcriptomes of *A. aegypti* (Matthews, et al. 2016), we used the published tool kallisto (Bray, et al. 2016). We first built an index using the *index* command on the complete transcriptome (TSA ID GFNA01). We then downloaded each set of reads from the NCBI SRA repository for the bioproject (PRJNA236239) and applied the quantification *quant* command to each individual dataset. Finally, we aggregated the results with the bioproject metadata to generate the final results. We also compared our results to the published quantification of transcripts done by the authors of the original study (Matthews, et al. 2016).

Analysis of *Gryllus bimaculatus* oskar transcript levels

The RNA-seq libraries of different tissues of *Gryllus bimaculatus* tissues were recently generated in our laboratory (Whittle, Kulkarni, et al. 2021b) and are available at NCBI (PRJNA564136). Cutadapt v3.4 (Martin 2011) was used to remove adapters and reads shorter than 20 nucleotides from the original fastq files. The gene expression in each library was quantified in transcripts per million (TPM) with RSEM v1.2.29 (Li and Dewey 2011), mapping the reads with STAR v2.7.0e1 (Dobin, et al. 2013) against the *G. bimaculatus* genome (Ylla, et al. 2021).

The *G. bimaculatus* gene GBI_01840 was identified as the putative locus coding for the previously reported *G. bimaculatus* Oskar protein (AFV31610.1) (Ewen-Campen, et al. 2012). Therefore, the TPM corresponding to the gene GBI_01840 in each library were considered to represent *oskar* mRNA expression in each of the sequenced libraries.

Semi-quantitative RT-PCR assessment of tissue-level *oskar* expression

Study species were as follows: live *Drosophila melanogaster* (Oregon R; Bloomington Stock Center #5); live *Callosobruchus maculatus* (Carolina Biological #144180); 100% ethanol-preserved *Aretaon asperrimus* (kind gift from Thies Büscher and Stanislav Gorb, Kiel University). We manually dissected ovaries, testes (including seminal vesicles), and whole heads from each specimen in 1X PBS. For *C. maculatus*, we also collected mixed-stage embryos for analysis as follows: adult female weevils were placed on a plate of fresh mung beans (Rani) and allowed to lay eggs for 24 hours. Embryos were removed from the beans by shaking beans with a wash solution (0.06M NaCl (VWR), 0.15% Triton X-100 (VWR), 50% bleach (Clorox) in distilled water) for 10 minutes. Embryos were collected in a basket and then rinsed with distilled water several times. Eggs and dissected tissues were transferred to TRIzol (Invitrogen) for RNA extraction. Total RNA was extracted using the manufacturer's protocol, including treatment of the RNA with DNase (Ambion) for 30 minutes at 37°C to remove genomic DNA. cDNA was synthesized using SuperScript III (Invitrogen). The amount of RNA used for DNase treatment and cDNA synthesis was standardized for tissues of a given species using a Nanodrop spectrophotometer. PCR was conducted with the resulting cDNA templates using Phusion polymerase (New England Biolabs) and the following PCR program: 98°C for 3 min; 35 cycles of 98°C for 30 sec, 70°C for 30 sec, 72°C for 30 sec (*D. melanogaster, C. maculatus*) or 1 min (*A. asperrimus*); and 72°C for 10 min. Primers were designed for both *oskar* and a housekeeping control gene for each species (see sequences of primers in table below). A pair of primers for each species were designed to span multiple exons, when possible, to test for genomic DNA contamination; no such contamination was detected. Reactions were run on 1.25% agarose gels

with a 1Kb plus DNA ladder (Invitrogen), and DNA products were visualized with Apex Safe DNA Gel Stain (Genesee Scientific).

**Gene**	**Forward Primer**	**Reverse Primer**	**Product Size**
Dmel oskar 1	ATGACGCCCACGCCAACGATTT	GCAATCAAATCGCACCACGCCC	534 bp
Dmel oskar 2	TTGCTGAGCCACGCCCAGAATG	GGCGGTTTTCAGTCGGTTCGGT	628 bp
Dmel RpL32	CACCAGTCGGATCGATATGC	CGATCCGTAACCGATGTTG	120 bp
Cmac oskar 1	GCTCTCAAAAGTGGCCAAAAACGA	ACTGCCAGCACAAATGACTTCA	700 bp
Cmac oskar 2	TGTGCTGGAAAAGTGCCACATGT	TAGGCAGCTTGGGAGACGGTGG	505 bp
Cmac RpL32	CAACTGCTGAGCACGTTCCACA	GGGTGAGAAGGCGCTTCAAGGG	224 bp
Aasp oskar 1	CGTCTCTTGCGTGGGCCATCAG	GCCGATGACTTGCCCGTTCCTC	380 bp
Aasp oskar 2	GAGGAACGGGCAAGTCATCGGC	ACTTCTTCACGCGGTGCAAGCA	294 bp
Aasp oskar 3	GGGAGGAGTTCCATTAGCACGGA	AGGCCTGGAAACTTTTGGCGGT	532 bp
Aasp RpL32	GCCATCTGTGGGTTACGGCAGC	GCTACGCAGGCGAGCATTTGCA	229 bp

Supplementary Figure Legends

Supplementary Figure S1: Summary statistics of the search for *oskar* orthologs. (a) Summary of searches and results for each of the three sources of data searched, from left to right: (i) The total number of datasets searched from all three sources (TSA: Transcriptome Shotgun Assembly Database; GCA: GenBank; GCF: RefSeq); (ii) the number of filtered *oskar* sequences identified in each of those datasets; and (iii) the proportion of filtered *oskar* sequences identified in each of the three sources. **(b)** Summary statistics broken down by insect orders. Only orders where an *oskar* sequence was identified are shown. From left to right: (iv) The number of *oskar* sequences identified in each of the three data sources; (v) the total number of filtered *oskar* sequences identified per order; (vi) the proportion of all searched datasets per order where an *oskar* sequences was identified. See also Supplementary Table 1

Supplementary Figure S2: Genome and transcriptome quality correlation to *oskar* identification. Shown are box plots of the distribution of *oskar* orthologs identified (ortholog identified or not identified) with respect to multiple genome and transcriptome quality metrics. For each metric, the means of both distributions were tested for significant differences using a Mann Whitney U test. A bar with an * is displayed if the p-value was less than 0.05. Mean and median values presented in Supplementary Table S2.

Supplementary Figure S3: Evidence for loss of *oskar* in Lepidoptera. Phylogeny of the Lepidoptera as per (Kawahara, et al. 2019). Next to each lepidopteran family are shown summary data regarding the status of *oskar* identification in our searches. Symbols with column labels in order from left to right: (i) vertical rectangles: grey: no *oskar* ortholog was identified in this family; range: at least one *oskar* ortholog was identified in this order. (ii) number of datasets searched. (iii) horizontal rectangles: proportion of searched datasets in which an *oskar* ortholog was identified; colors as in (i); numbers and proportions at right. (iv) pie chart: proportion of *oskar* sequences identified in RefSeq (GCF) datasets; numbers and proportions at right. (v) pie chart: proportion of *oskar* sequences identified in GenBank (GCA) datasets; numbers and proportions at right. (vi) pie chart: proportion of *oskar* sequences identified in Transcriptome Shotgun Assembly Database (TSA) datasets; numbers and proportions at right. Circles to the right of some family names indicate that there is literature evidence for involvement of germ plasm (black) or no germ plasm (white) in germ cell specification. Numbers to the left of the circles indicate references to the primary literature as follows: [1]: (Kobayashi and Ando 1984); [2] (Ando and Tanaka 1979); [3] (Lautenschlager 1932); [4] (Anderson and Wood 1968); [5] (Tanaka 1987); [6] (Woodworth 1889); [7] (Eastham 1930); [8] (Berg and Gassner 1978); [9-10] (Sehl 1931; Guelin 1994); [11] (Johannsen 1929); [12] (Presser and Rutschky 1957); [13-23]: (Tomaya 1902; Schwangart 1905; Saito 1937; Miya 1953, 1958, 1975; Nakao 1999; Toshiki, et al. 2000; Nakao, et al. 2006; Nakao, et al. 2008; Nakao and Takasu 2019). No datasets were available for Urudidea, Sesidea, Alucitidea, Callidulidea, Mimallonidea, Drepanidea or Lasiocampidea at the time of analysis.

Supplementary Figure S4: Evidence for duplication of *oskar* in Hymenoptera. Phylogenetic tree of all hymenopteran Oskar sequences inferred using RaxML with 100 bootstraps. Branch length normalized to show only the topology. Each leaf is an Oskar ortholog. Gray: only one Oskar sequence was identified in this species. Red: putatively duplicated Oskar sequences (sequence

similarity < 80%; see Methods). Families containing *oskar* duplications are highlighted as per Figure 4.

Supplementary Figure S5: Tissue-level *oskar* expression in a mosquito. *oskar* transcripts per million (TPM) in different mosquito tissues based on data reported in REF. (a, b) Quantified from the raw reported data using kallisto (Bray, et al. 2016) and (c, d) using the original study's quantification results (Matthews, et al. 2016). Error bars show the standard error of at least three independent measurements. All tissues reported in the study that were not brain, ovaries or abdominal tips were placed in the “other tissues” category. As Matthews and colleagues (2016) performed RNA sequencing experiments on female mosquitoes before and after a blood meal, (b) and (d) show the impact of the two different feedings conditions on *oskar* expression in the abdominal tip and ovaries.

Supplementary Figure S6: Tissue-level *oskar* expression in a cricket. Transcripts per million (TPM) of *oskar* in each tissue of *G. bimaculatus* for which RNA-seq was generated by Whittle, Kulkarni, et al. (2021b). For each tissue type, either two or three biological samples were sequenced as replicates. Each dot represents the expression level of *oskar* in a single biological sample.

Supplementary Figure S7: Tissue-level *oskar* expression in a fly, a weevil and a stick insect. Three study species were selected to evaluate tissue-level *oskar* expression patterns utilizing RT-PCR: *Drosophila melanogaster* (a), *Callosobruchus maculatus* (b), and *Aretaon asperrimus* (c). The top of each figure shows a schematic of the *oskar* transcript with exons marked if known (not drawn to scale). Approximate primer locations for PCR products are marked with arrows below each schematic. Gel images show RT-PCR products for the amplified *oskar* regions and a housekeeping control gene. Templates for these reactions included cDNA synthesized from male (m) and female (f) adult head and gonads, as well as from 0-24 hour embryos for *C .maculatus*. Water was used as a negative control.

Supplementary Figure S8: Tissue and developmental stage metadata analysis of *oskar* identification in transcriptome datasets. (a) Proportion of analyzed datasets that were sequenced from the developmental stages indicated on the Y axis. **(b)** Proportion of analyzed datasets per developmental stage in which an *oskar* ortholog was identified (red). **(c)** Proportion of analyzed datasets that were sequenced from the tissue type indicated on the Y axis. **(d)** Proportion of analyzed datasets per tissue type in which an *oskar* ortholog was identified (red)

Supplementary Figure S9: Evolution of the structure of Oskar in Diptera. Left: dipteran phylogeny from (Maddison, et al. 2007; Wiegmann, et al. 2011). Top: schematic representation of Oskar domain structure. Blue: heatmap showing the overall occupancy of an amino acid position in the Oskar alignment trimmed for at least 10% overall occupancy at a given position. For each dipteran family, occupancy at a given position is defined as (number of non-gap amino acids / number of sequences in that family). If a 3’ or 5’ extension (defined as a coding sequence unbroken by stop codons, 5’ of the first residue of the LOTUS domain, or 3’ of the last residues of the OSK domain but 5’ to a predicted poly-A tail) was detected in a family, a black box outlines the putative domain. Any such identified 5’ domains were designated as putative “Long Oskar” domains.

Supplementary Figure S10: Multiple Correspondence Analysis (MCA) of full-length Oskar, the OSK domain and the LOTUS domain. MCA analysis of trimmed (30% occupancy) alignments for (a) full-length Oskar, (b) the OSK domain and (c) the LOTUS domain colored by insect order (see legend at right). The alignment was projected onto the first three main MCA dimensions (1, 2 and 3). Each dot corresponds to one sequence. Dotted line outlines specific families of interest as discussed in the text

Supplementary Figure S11: Oskar domains secondary structure conservation. Sequence Logo of Jpred4 predictions for LOTUS and OSK domains showing the conservation of secondary structures, computed with WebLogo (Crooks, et al. 2004). The height of each letter represents that state's (X, H or B) conservation throughout the alignment in bits. X (black): unfolded amino acids; H (red): α helices; E (blue): β sheets. **(a)** Prediction for the LOTUS domain. **(b)** Prediction for the OSK domain.

Supplementary Figure S12: Duplications and losses of *oskar* in Hymenoptera. Absence (magenta) or presence of *oskar* orthologs detected in single copy (cyan) or multiple copies (yellow) in the genomic or transcriptomic datasets examined in this study. Genera shown in italics indicate individual species searched and are abbreviated simply for space reasons. Genera shown in regular type (not italics) indicate a summary of the results from multiple congeneric species, which were nearly always consistent within genera; in all cases where intrageneric results for *oskar* presence or absence were inconsistent, we gave precedence for the finding obtained from a genome sequence (GCF or GCA) over findings obtained from a transcriptome (TSA)), o for Hymenoptera species or genera. For some species, germ cell specification via germ plasm (black circles) or differentiation from mesoderm (no germ plasm; white circles) has been reported in the literature, with primary data references indicated by numbers as follows: [1-6]: (Bütschli 1870; Fleig and Sander 1985, 1986; Zissler 1992; Gutzeit, et al. 1993; Dearden 2006); [7]: (Khila and Abouheif 2008); [8-10]: (Bull 1982; Lynch and Desplan 2010; Lynch, et al. 2011); [11]: (Koscielska and Koscielski 1987); [12-13]: (Silvestri 1906, 1908); [12, 14-21]: (Silvestri 1906; Hegner 1914; Grbic', et al. 1996; Strand and Grbic' 1997; Grbic' 2000, 2003; Donnell, et al. 2004; Zhurov, et al. 2004); [22-25]: (Gatenby 1917a; Gatenby 1917b; Gatenby 1918; Gatenby 1920); [24]: (Amy 1961); [27-28]: (Gatenby 1920; Tawfik 1957); [29-30]: (Bronskill 1959; Fleischmann 1975); [31]: (Shafiq 1954); [32]: (Sumitani, et al. 2003). Phylogenetic relationships as per (Nyman, et al. 2006; Field, et al. 2011; Schmidt 2013; Prous, et al. 2014; Ward 2014; Malm and Nyman 2015; Vilhelmsen 2015; Ward, et al. 2016; Peters, et al. 2017; Chen and Achterberg 2018; Peters, et al. 2018; Sharanowski, et al. 2021). Evolution of major hymenopteran life history characteristics (eusociality, pollen collecting, stinger, parasitoidism) as per (Peters, et al. 2017).

Supplementary References

Amy RL. 1961. The embryology of *Habobracon juglandis* (Ashmead). Journal of Morphology 109:199-217.

Anderson DT, Wood EC. 1968. The morphological basis of embryonic movements in the light brown apple moth, *Epiphyas postvittana* (Walk.) (Lepidoptera, Tortricidae). Australian Journal of Zoology 16:763-793.

Ando H, Tanaka M. 1979. Early embryonic devekopment of the primitive moths, *Enduclyta signifer* Walker and *E. excrescens* Butler (Lepidoptera: Hepialidae). International Journal of Insect Morphology and Embryology 9:67-77.

Berg GJ, Gassner G. 1978. Fine structure of the blastoderm embryo of the pink bollworm, *Pectinophora gossypiella* (Saunders) (Lepidoptera: gelechiidae). International Journal of Insect Morphology and Embryology 1:81+105.

Bray NL, Pimentel H, Melsted P, Pachter L. 2016. Near-optimal probabilistic RNA-seq quantification. Nature Biotechnology 34:525-527.

Bronskill JF. 1959. Embryology of *Pimpla turionellae* (L.) (Hymenoptera: Ichneumonidae). Canadian Journal of Zoology 37:655-688.

Bull AL. 1982. Stages of living embryos in the jewel wasp *Mormoniella (Nasonia) vitripennis* (Walker) (Hymenoptera: Pteromalidae). International Journal of Insect Morphology and Embryology 11:1-23.

Bütschli O. 1870. Zur Entwicklungsgeschichte der Biene. Zeitschrift für Wissenschaftliche Zoologie 20:519-564.

Chen X-x, Achterberg Cv. 2018. Systematics, Phylogeny, and Evolution of Braconid Wasps: 30 Years of Progress. Annual Review of Entomology 64:1-24.

Crooks GE, Hon G, Chandonia JM, Brenner SE. 2004. WebLogo: a sequence logo generator. Genome Research 14:1188-1190.

Dearden PK. 2006. Germ cell development in the Honeybee (*Apis mellifera*); *vasa* and *nanos* expression. BMC Developmental Biology 6:6.

Dobin A, Davis CA, Schlesinger F, Drenkow J, Zaleski C, Jha S, Batut P, Chaisson M, Gingeras TR. 2013. STAR: ultrafast universal RNA-seq aligner. Bioinformatics 29:15-21.

Donnell DM, Corley LS, Chen G, Strand MR. 2004. Caste determination in a polyembryonic wasp involves inheritance of germ cells. Proceedings of the National Academy of Sciences of the United States of America 101:10095-10100.

Eastham LES. 1930. The embryology of *Pieris rapae* - Organogeny. Philosophical Transactions of the Royal Society of London. Series B: Biological Sciences 219:1-50.

Ewen-Campen B, Srouji JR, Schwager EE, Extavour CG. 2012. Oskar predates the evolution of germ plasm in insects. Curr Biol 22:2278-2283.

Field J, Ohl M, Kennedy M. 2011. A molecular phylogeny for digger wasps in the tribe Ammophilini (Hymenoptera, Apoidea, Sphecidae). Systematic Entomology 36:732-740.

Fleig R, Sander K. 1985. Blastoderm development in honey bee embryogenesis as seen in the scanning electron microscope. International Journal of Invertebrate Reproduction and Development 8:279-286.

Fleig R, Sander K. 1986. Embryogenesis of the Honeybee *Apis mellifera* L (Hymenoptera, Apidae) - an SEM Study. International Journal of Insect Morphology and Embryology 15:449-462.

Fleischmann VG. 1975. Origin and embryonic development of fertile gonads with and without pole cells of *Pimpla turionellae* L. (Hymenoptera, Ichneumonidae). Zool. Jb. Anat. Bd. 94:375-411.

Gatenby JB. 1920. The Cytoplasmic Inclusions of the Germ Cells. Part VI. On the origin and probable constitution of the germ-cell determinant of *Apanteles glomeratus,* with a note on the secondary nuclei. Quarterly Journal of Microscopical Science 64:133-153.

Gatenby JB. 1917a. The embryonic development of *Trichogramma evanescens* Westw., monoembryonic egg parasite of *Donacia simplex*. Quarterly Journal of Microscopical Science 62:149-187.

Gatenby JB. 1918. The segregation of germ cells in *Trichogramma evanescens*. Quarterly Journal of Microscopical Science 63:161-173.

Gatenby JB. 1917b. The segregation of the germ-cells in *Trichogramma evanescens*. Quarterly Journal of Microscopical Science 62:149-187.

Grbic' M. 2000. "Alien" wasps and evolution of development. Bioessays 22:920-932.

Grbic' M. 2003. Polyembryony in parasitic wasps: evolution of a novel mode of development. International Journal of Developmental Biology 47:633-642.

Grbic' M, Nagy LM, Carroll SB, Strand M. 1996. Polyembryonic development: insect pattern formation in a cellularised environment. Development:795-804.

Guelin M. 1994. [Activity of W-sex heterochromatin and accumulation of the nuage in nurse cells of the lepidopteran *Ephestia*]. C. R. Acad. Sci. Paris. Ser. III 317:54-61.

Gutzeit HO, Zissler D, Fleig R. 1993. Oogenesis in the Honeybee *Apis mellifera* - Cytological Observations on the Formation and Differentiation of Previtellogenic Ovarian Follicles. Rouxs Archives of Developmental Biology 202:181-191.

Hegner RW. 1914. Studies on germ cells. III. The origin of the Keimbahn-determinants in a parasitic Hymenopteran, *Copidosoma*. Anatomischer Anzeiger 3-4:51-69.

Johannsen OA. 1929. Some phases in the embryonic development of *Diacrisia virginica* Fabr. (Lepidoptera). J. Morphol. Physiol. 2:493-541.

Kawahara AY, Plotkin D, Espeland M, Meusemann K, Toussaint EFA, Donath A, Gimnich F, Frandsen PB, Zwick A, Dos Reis M, et al. 2019. Phylogenomics reveals the evolutionary timing and pattern of butterflies and moths. Proceedings of the National Academy of Sciences of the United States of America 116:22657-22663.

Khila A, Abouheif E. 2008. Reproductive constraint is a developmental mechanism that maintains social harmony in advanced ant societies. Proceedings of the National Academy of Sciences of the United States of America 105:17884-17889.

Kobayashi Y, Ando H. 1984. Mesodermal Organogenesis in the Embryo of the Primitive Moth, *Neomicropteryx nipponensis* Issiki (Lepidoptera, Micropterygidae). Journal of Morphology 181:29-47.

Koscielska MK, Koscielski B. 1987. Early embryonic development of *Tritneptis diprionis* (Chalcidoidea, Hymenoptera). In: Ando H, Jura C, editors. Recent Advances in Insect Embryology in Japan and Poland. Tsukuba: Arthropod. Embryol. Soc. Jpn.

ISEBU Co. Ltd. p. 207-214.

Laurence BR. 1977. Ovary development in mosquitoes: a review. Adv. Invertebr. Repr. 1:154-165.

Lautenschlager F. 1932. Die Embryonalentwicklung der weiblichen Keimdruse bei der Psychide *Solenobia triquetella*. Zool. Jarh. 56:121-162.

Leader DP, Krause SA, Pandit A, Davies SA, Dow JAT. 2017. FlyAtlas 2: a new version of the Drosophila melanogaster expression atlas with RNA-Seq, miRNA-Seq and sex-specific data. Nucleic Acids Research 46:gkx976-.

Li B, Dewey CN. 2011. RSEM: accurate transcript quantification from RNA-Seq data with or without a reference genome. BMC Bioinformatics 12:323.

Lynch JA, Desplan C. (p16123 co-authors). 2010. Novel modes of localization and function of *nanos* in the wasp *Nasonia*. Development 137:3813-3821.

Lynch JA, Özüak O, Khila A, Abouheif E, Desplan C, Roth S. 2011. The Phylogenetic Origin of *oskar* Coincided with the Origin of Maternally Provisioned Germ Plasm and Pole Cells at the Base of the Holometabola. PLoS Genetics 7:e1002029.

Maddison DR, Schultz K-S, Maddison WP. 2007. The Tree of Life Web Project. Zootaxa 1668:19-40.

Malm T, Nyman T. 2015. Phylogeny of the symphytan grade of Hymenoptera: new pieces into the old jigsaw(fly) puzzle. Cladistics 31:1-17.

Martin M. 2011. Cutadapt removes adapter sequences from high-throughput sequencing reads. EMBnet. journal 17:10-12.

Matthews BJ, McBride CS, DeGennaro M, Despo O, Vosshall LB. 2016. The neurotranscriptome of the Aedes aegypti mosquito. BioMedCentral Genomics 17:32.

Miya K. 1953. The presumptive genital region at the blastoderm stage of the silkworm egg. Journal of the Faculty of Agriculture of Iwate University:223-227.

Miya K. 1958. Studies on the embryonic development of the gonad in the silkworm, *Bombyx mori* L. Part I. Differentiation of germ cells. Journal of the Faculty of Agriculture of Iwate University 3:436-467.

Miya K. 1975. Ultrastructural changes of embryonic cells during organogenesis in the silkworm, *Bombyx mori*. I. The Gonad. Journal of the Faculty of Agriculture of Iwate University 12:329-338.

Nakao H. 1999. Isolation and characterization of a *Bombyx vasa*-like gene. Development Genes and Evolution 209:312-316.

Nakao H, Hatakeyama M, Lee JM, Shimoda M, Kanda T. 2006. Expression pattern of *Bombyx* vasa-like (BmVLG) protein and its implications in germ cell development. Development Genes and Evolution 216:94-99.

Nakao H, Matsumoto T, Oba Y, Niimi T, Yaginuma T. 2008. Germ cell specification and early embryonic patterning in Bombyx mori as revealed by nanos orthologues. Evolution and Development 10:546-554.

Nakao H, Takasu Y. 2019. Complexities in Bombyx germ cell formation process revealed by Bm-nosO (a Bombyx homolog of nanos) knockout. Developmental Biology 445:29-36.

Nyman T, Zinovjev AG, Vikberg V, Farrell BD. 2006. Molecular phylogeny of the sawfly subfamily Nematinae (Hymenoptera: Tenthredinidae). Systematic Entomology 31:569-583.

Peters RS, Krogmann L, Mayer C, Donath A, Gunkel S, Meusemann K, Kozlov A, Podsiadlowski L, Petersen M, Lanfear R, et al. 2017. Evolutionary History of the Hymenoptera. Current Biology 27:1013-1018.

Peters RS, Niehuis O, Gunkel S, Bläser M, Mayer C, Podsiadlowski L, Kozlov A, Donath A, Noort Sv, Liu S, et al. 2018. Transcriptome sequence-based phylogeny of chalcidoid wasps (Hymenoptera: Chalcidoidea) reveals a history of rapid radiations, convergence, and evolutionary success. Molecular Phylogenetics and Evolution 120:286-296.

Presser BD, Rutschky CW. 1957. The embryonic development of the corn earworm, *Heliothis zea* (Boddie) (Lepidoptea, Phalaenidae). Annals of the Entomological Society of America 50:133-164.

Prous M, Blank SM, Goulet H, Heibo E, Liston A, Malm T, Nyman T, Schmidt S, Smith DR, Vårdal H, et al. 2014. The genera of Nematinae (Hymenoptera, Tenthredinidae). Journal of Hymenoptera Research 40:1-69.

Saito. 1937. On the development of the Tusser, *Antheraea pernyi* Guerin-Meneville, with special reference to the comparative embryology of insects. Journal of the Faculty of Agriculture of Hokkaido Imperial University 40:35-109.

Schmidt C. 2013. Molecular phylogenetics of ponerine ants (Hymenoptera: Formicidae: Ponerinae). Zootaxa 3647:201-250.

Schwangart F. 1905. Zur Entwickslungsgeschichte der Lepidopteren. Biol. Centralbl. 25:777-789.

Sehl A. 1931. Furchung und Bildung der Keimanlage bei der Mehlmotte *Ephestia kuehniella*. Zell. Zeit. Morph. U. Okol. 1:429-506.

Shafiq SA. 1954. A study of the embryonic development of the Gooseberry Sawfly, *Pteronidea ribesii*. Quarterly Journal of Microscopical Science 95:93-114.

Sharanowski BJ, Ridenbaugh RD, Piekarski PK, Broad GR, Burke GR, Deans AR, Lemmon AR, Lemmon ECM, Diehl GJ, Whitfield JB, et al. 2021. Phylogenomics of Ichneumonoidea (Hymenoptera) and implications for evolution of mode of parasitism and viral endogenization. Molecular Phylogenetics and Evolution 156:107023.

Silvestri F. 1906. Contribuzioni alla conoscenza biologica degli Imenotteri parassiti. I. Biologia del *Litomastix truncellatus* Dalm. Annali della r. Scuola Superiore di Agricoltura in Portici 6:3-51.

Silvestri F. 1908. Contribuzioni alla conoscenza degli Imenotteri parassiti. Bollettino del Laboratorio di Zoologia Generale e Agraria della r. Scuola Superiore d'Agricoltura (AFTW. Facoltà Agraria) in Portici 3:29-84.

Strand MR, Grbic' M. 1997. The Development and Evolution of Polyembryonic Insects. Current Topics in Developmental Biology 35:121-159.

Sumitani M, Yamamoto DS, Oishi K, Lee JM, Hatakeyama M. 2003. Germline transformation of the sawfly, *Athalia rosae* (Hymenoptera: Symphyta), mediated by a piggyBac-derived vector. Insect Biochem Mol Biol 33:449-458.

Tanaka M. 1987. Differentiation and behaviour of Primordial Germ Cells during the Early Embryonic Development of *Parnassius glacialis* Butler, *Luehdorfia japonica* Leech and *Byasa (Atrophaneura) alcinous alcinous* Klug (Lepidoptera: Papilionidae). In: Ando H, Jura C, editors.

Recent Advances in Insect Embryology in Japan and Poland. Tsukuba: Arthropod. Embryol. Soc. Jpn.

ISEBU Co. Ltd. p. 255-266.

Tawfik MFS. 1957. Alkaline phosphatase in the germ-cell determinant of the egg of *Apanteles*. Journal of Insect Physiology 1:286-291.

Tomaya K. 1902. On the embryology of the silkworm. Bulletin of the College of Agriculture, Tokyo 5:73-111.

Toshiki T, Chantal C, R., Toshio K, Eappen A, Mari K, Natuo K, Jean-Luc T, Bernard M, Gérard C, Paul S, et al. 2000. Germline transformation of the silkworm *Bombyx mori* L. using a piggyBac transposon-derived vector. Nature Biotech.:81-84.

Vilhelmsen L. 2015. Morphological phylogenetics of the Tenthredinidae (Insecta:Hymenoptera). Invertebrate Systematics 29:164-190.

Ward PS. 2014. The Phylogeny and Evolution of Ants. Annual Review of Ecology, Evolution, and Systematics 45:23-43.

Ward PS, Blaimer BB, Fisher BL. 2016. A revised phylogenetic classification of the ant subfamily Formicinae (Hymenoptera: Formicidae), with resurrection of the genera Colobopsis and Dinomyrmex. Zootaxa 4072:343-357.

Whittle CA, Kulkarni A, Chung N, Extavour CG. 2021. Adaptation of codon and amino acid use for translational functions in highly expressed cricket genes. BioMedCentral Genomics 22.

Whittle CA, Kulkarni A, Extavour CG. 2021a. Evolutionary dynamics of sex-biased genes expressed in cricket brains and gonads. Journal of Evolutioary Biology doi:10.1111/jeb.13889.

Whittle CA, Kulkarni A, Extavour CG. 2021b. Evolutionary dynamics of sex-biased genes expressed in cricket brains and gonads. J Evol Biol 34:1188-1211.

Wiegmann BM, Trautwein MD, Winkler IS, Barr NB, Kim J-W, Lambkin C, Bertone MA, Cassel BK, Bayless KM, Heimberg AM, et al. (r40919 co-authors). 2011. Episodic radiations in the fly tree of life. Proceedings of the National Academy of Sciences 108:5690-5695.

Woodworth CW. 1889. Studies on the embryological development of *Euvanessa antiopa*. In: Scudder, editor. Butterflies of Eastern United States and Canada. p. 102.

Ylla G, Nakamura T, Itoh T, Kajitani R, Toyoda A, Tomonari S, Bando T, Ishimaru Y, Watanabe T, Fuketa M, et al. 2021. Insights into the genomic evolution of insects from cricket genomes. Commun Biol 4:733.

Zhurov V, Terzin T, Grbic M. 2004. Early blastomere determines embryo proliferation and caste fate in a polyembryonic wasp. Nature 432:764-769.

Zissler D. 1992. From egg to pole cells: ultrastructural aspects of early cleavage and germ cell determination in insects. Micr. Res. and Tech.:49-74.

464 **Supplementary Figure S1**

465

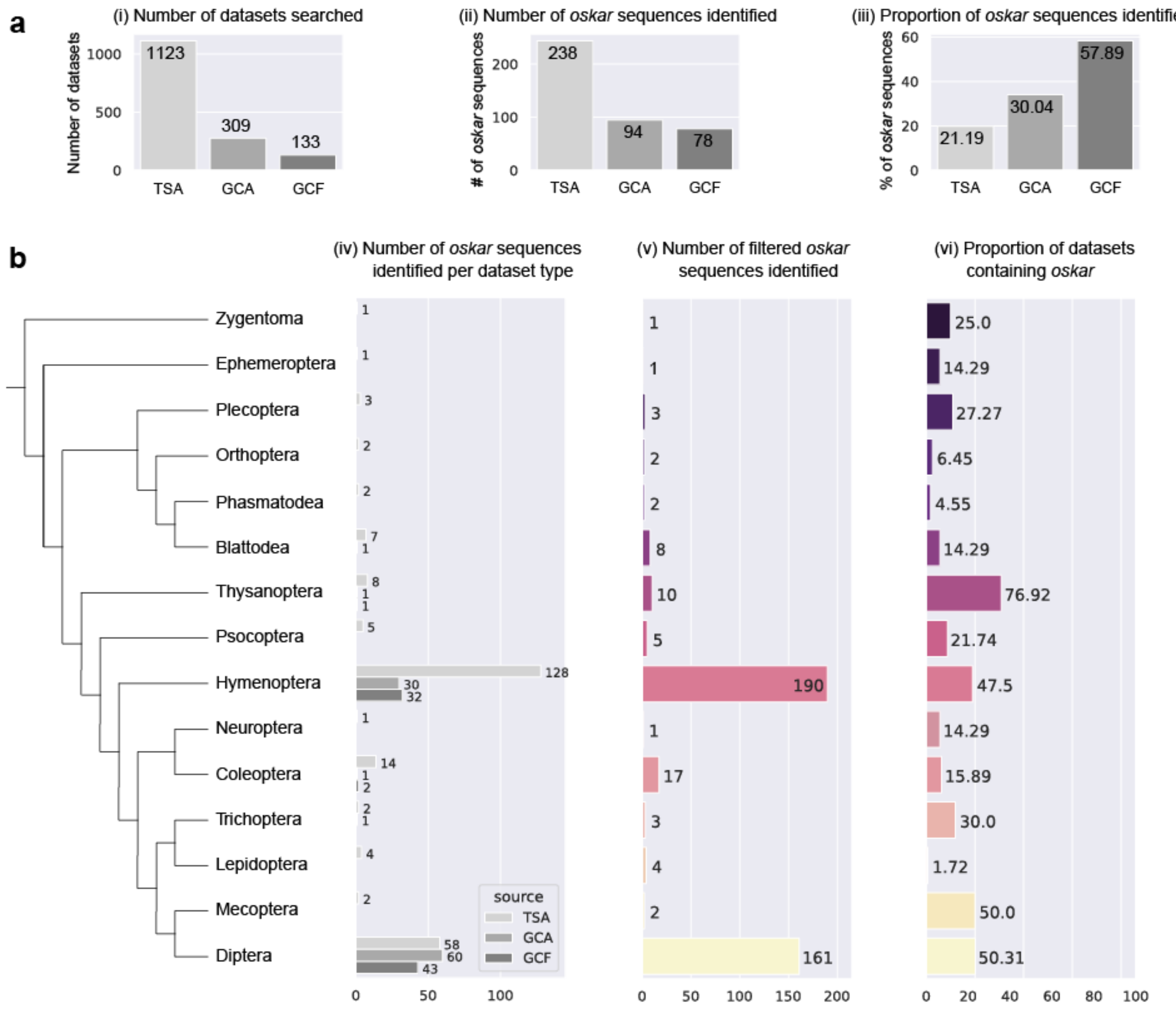

466

467 .

Supplementary Figure S2

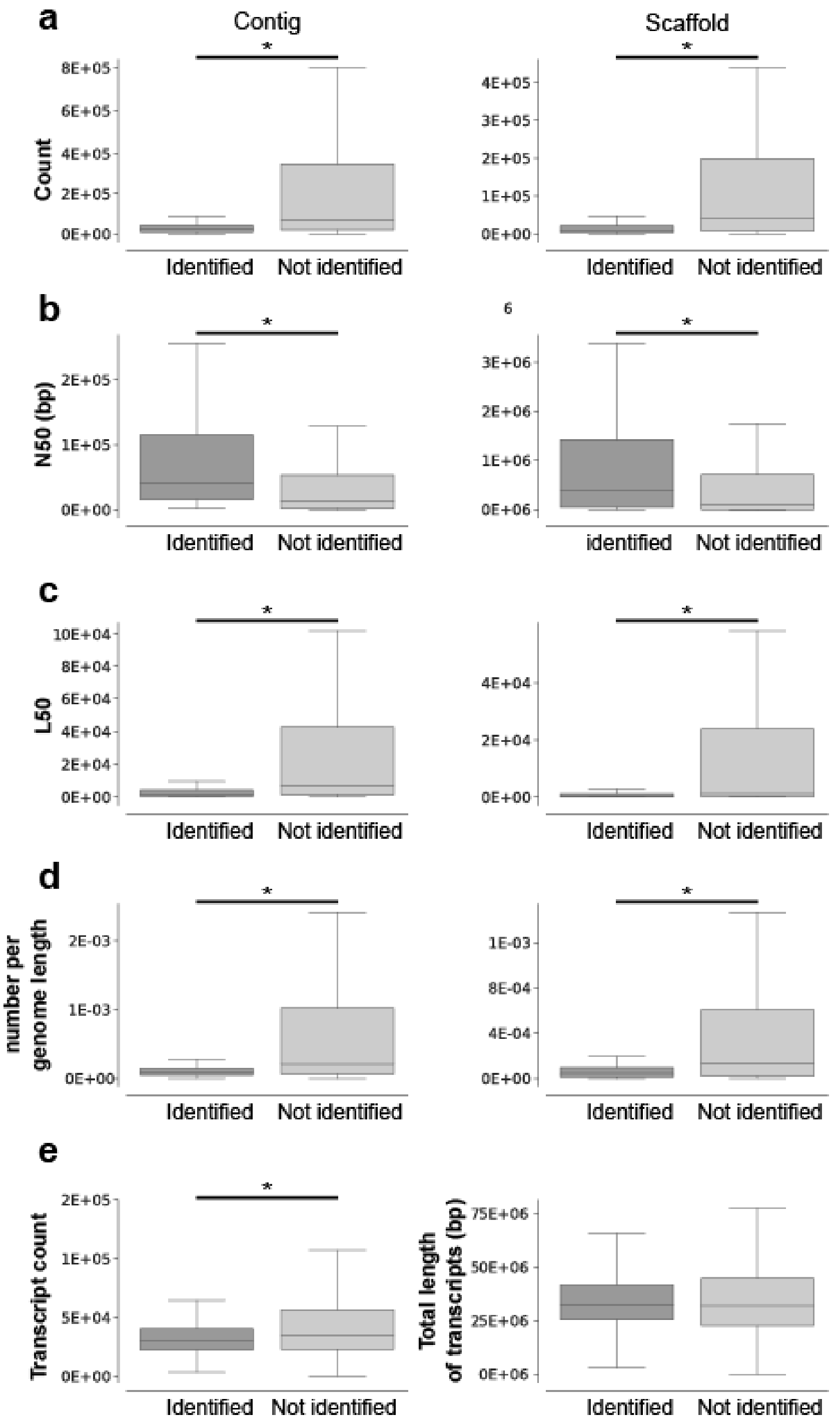

Supplementary Figure S3

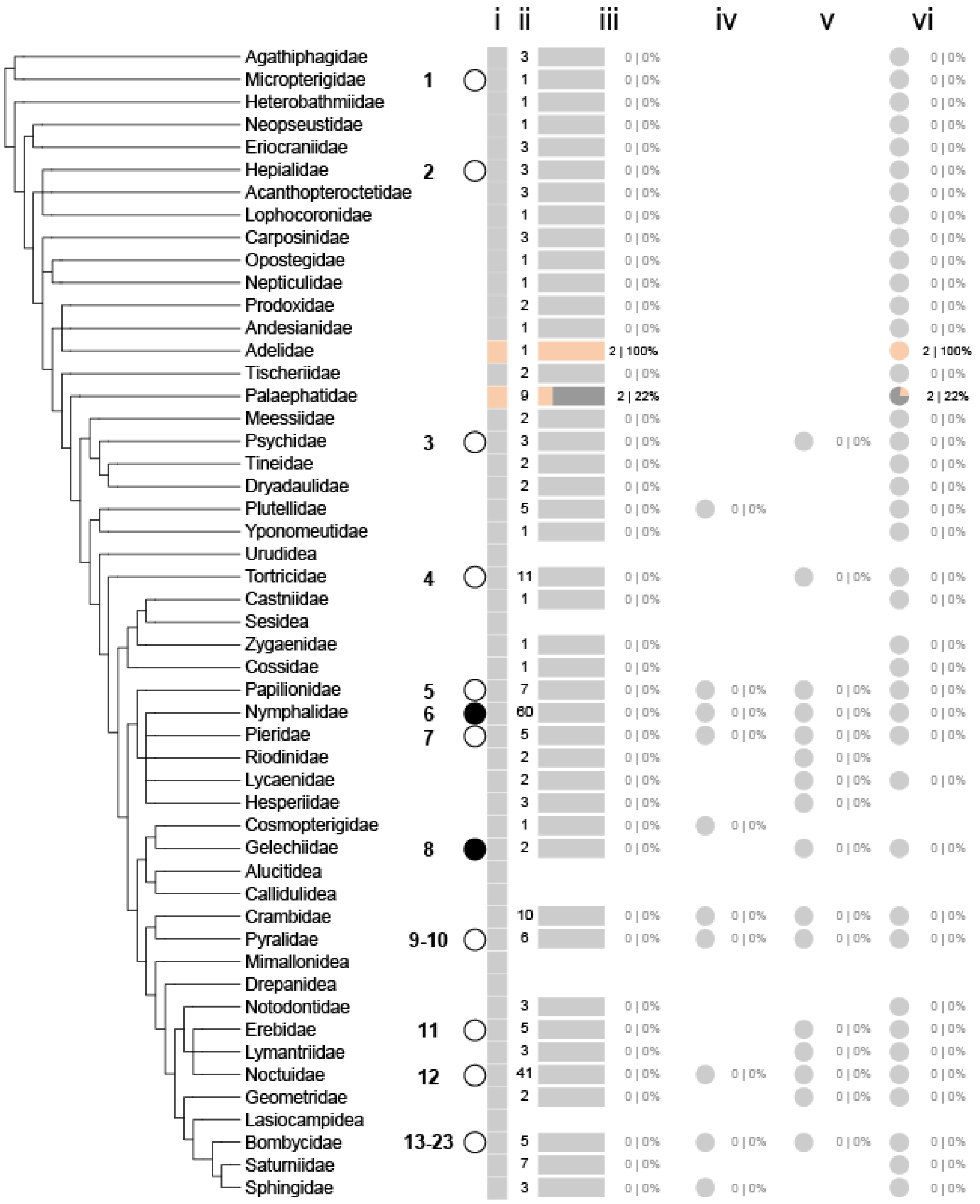

Supplementary Figure S4

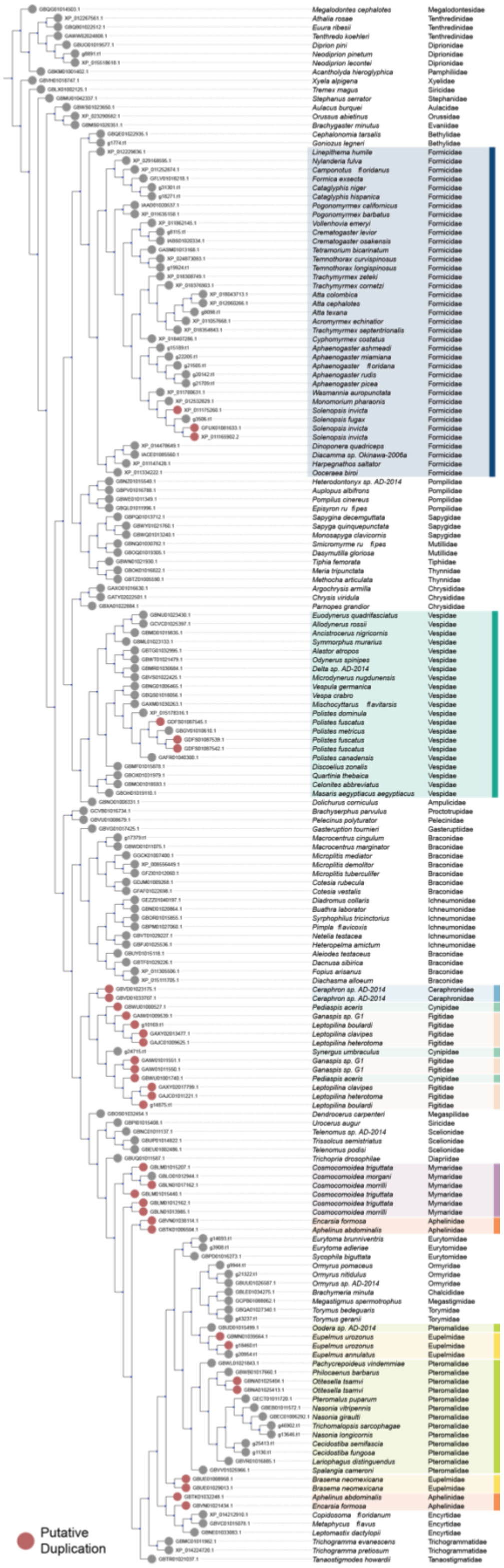

Supplementary Figure S5

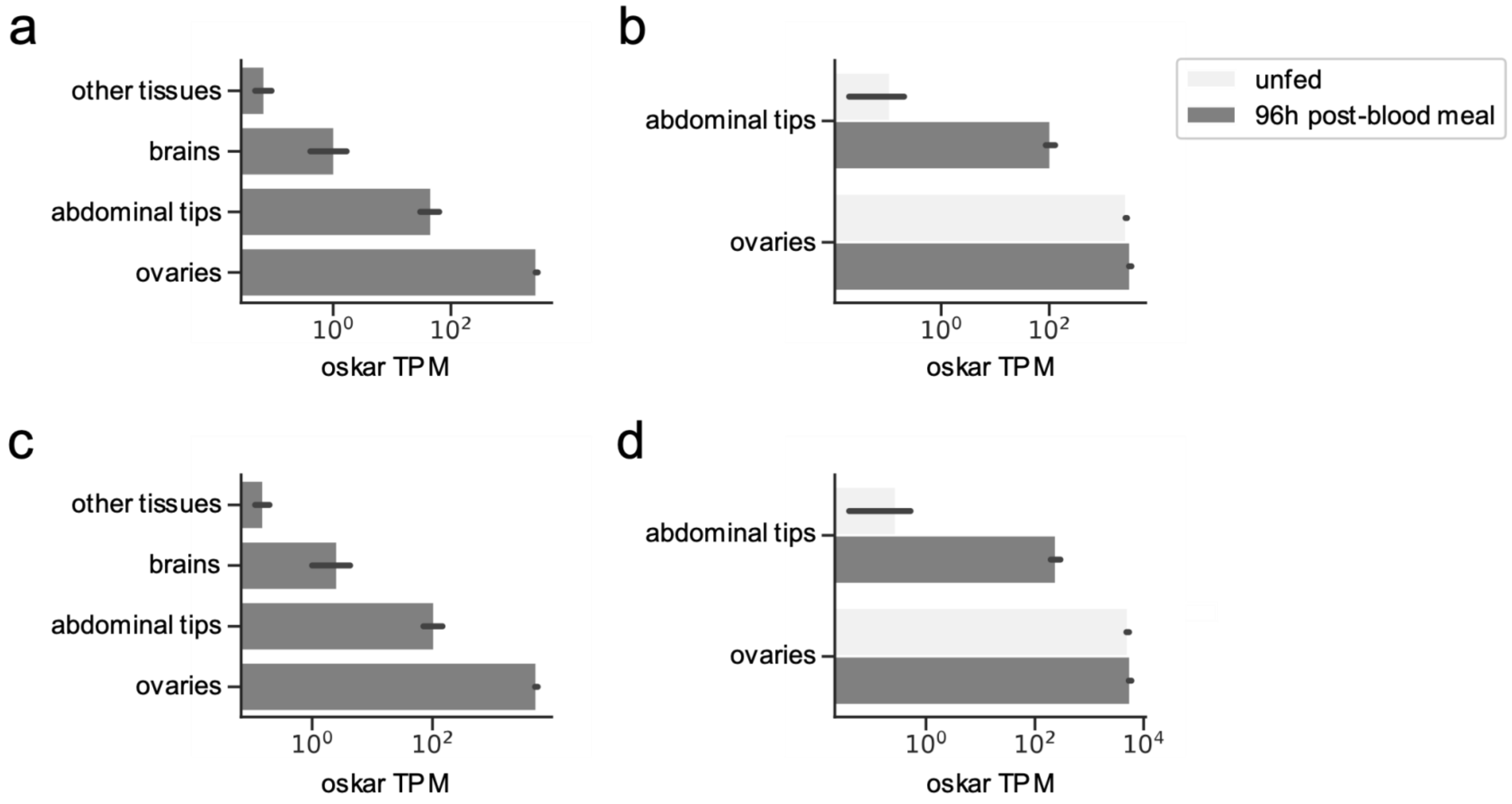

Supplementary Figure S6

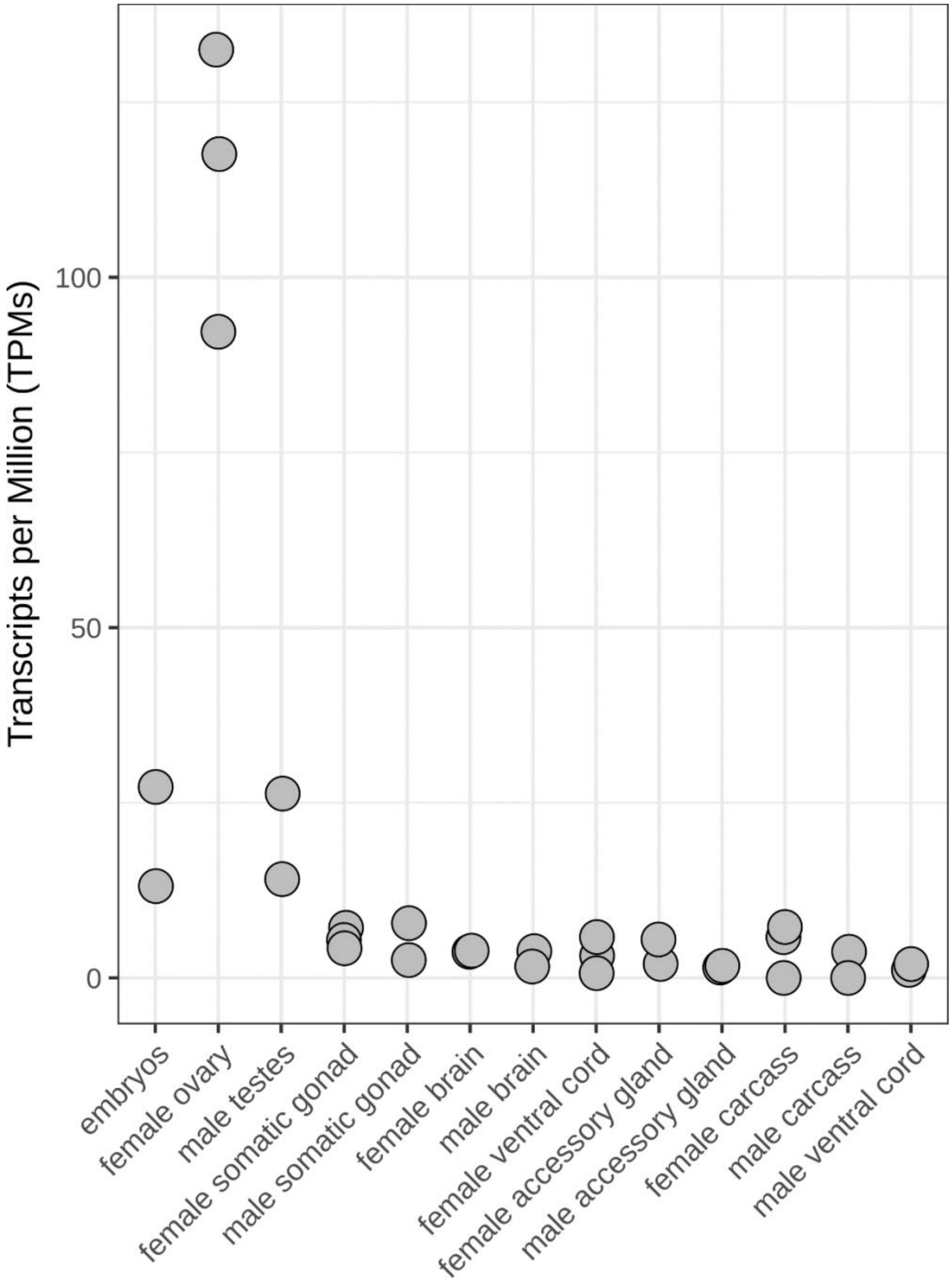

Supplementary Figure S7

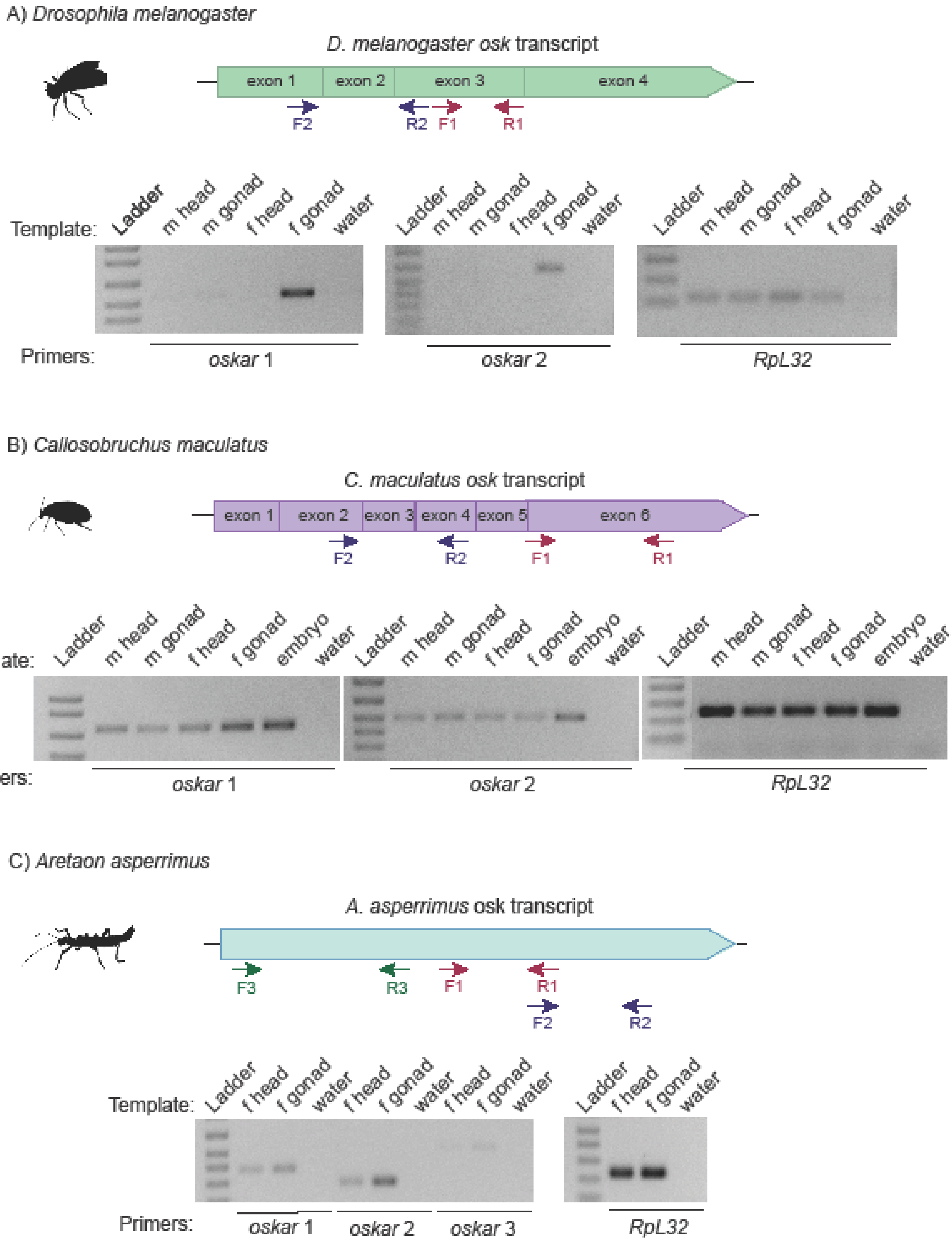

492 **Supplementary Figure S8**

493

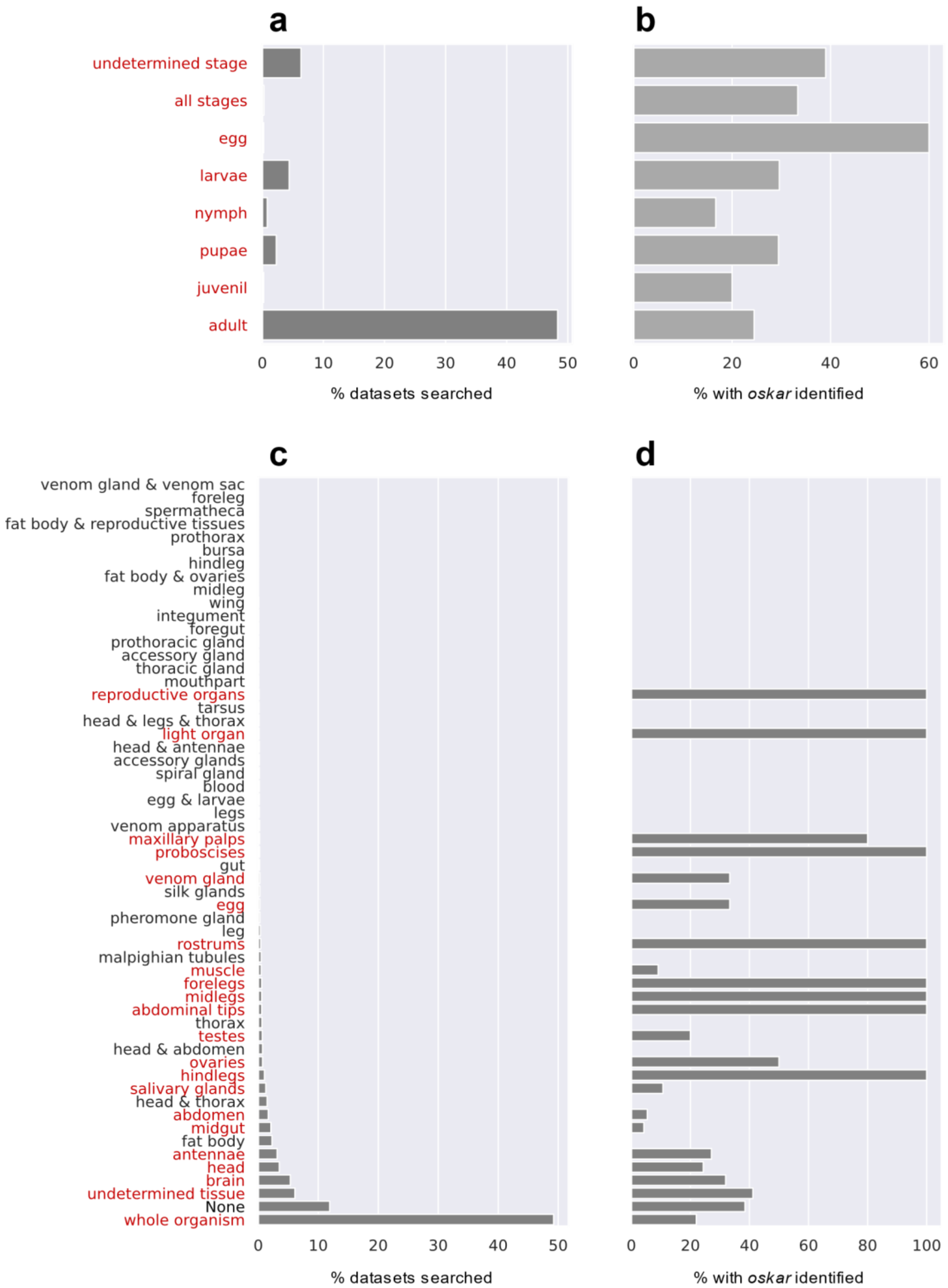

494

495

Supplementary Figure S9

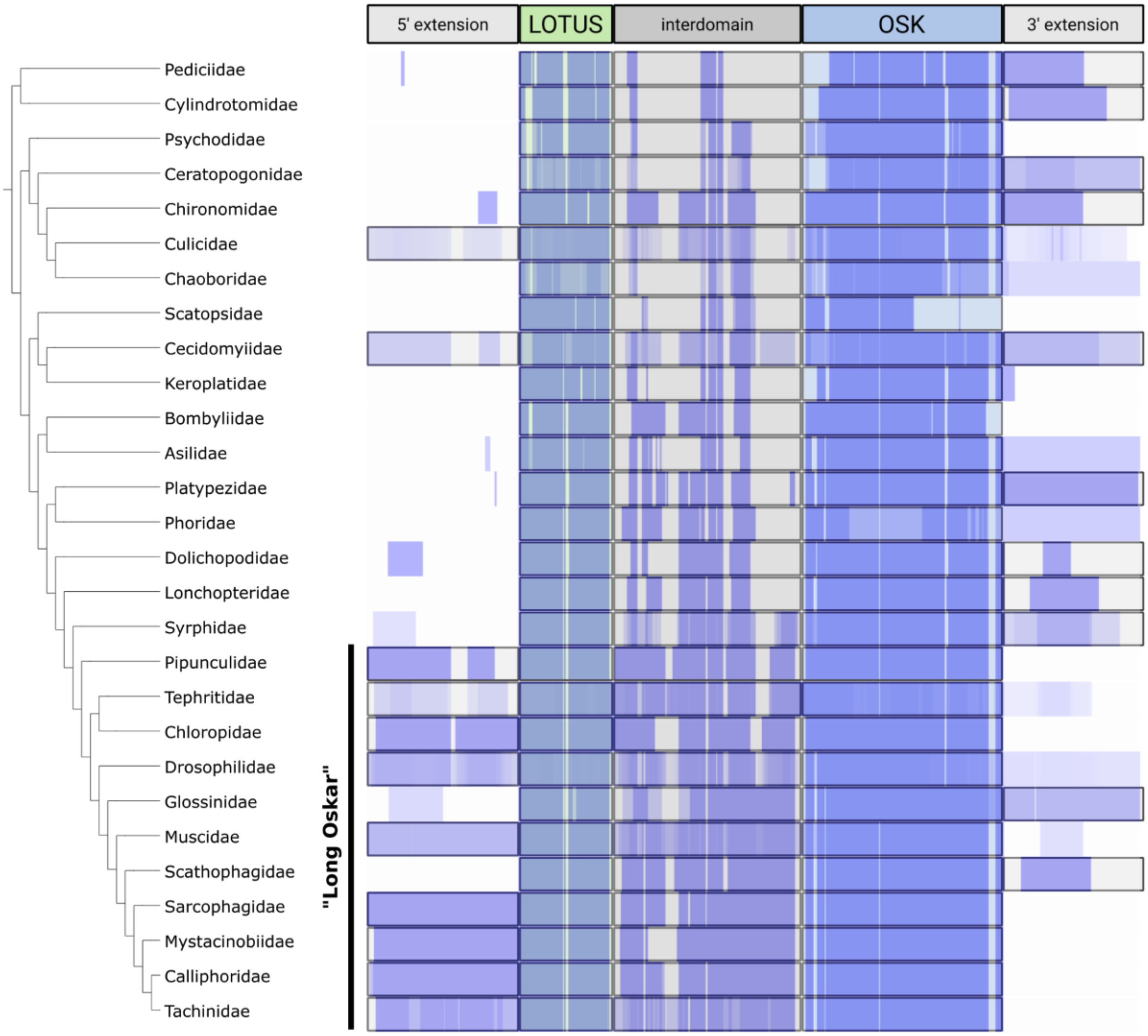

.

Supplementary Figure S10

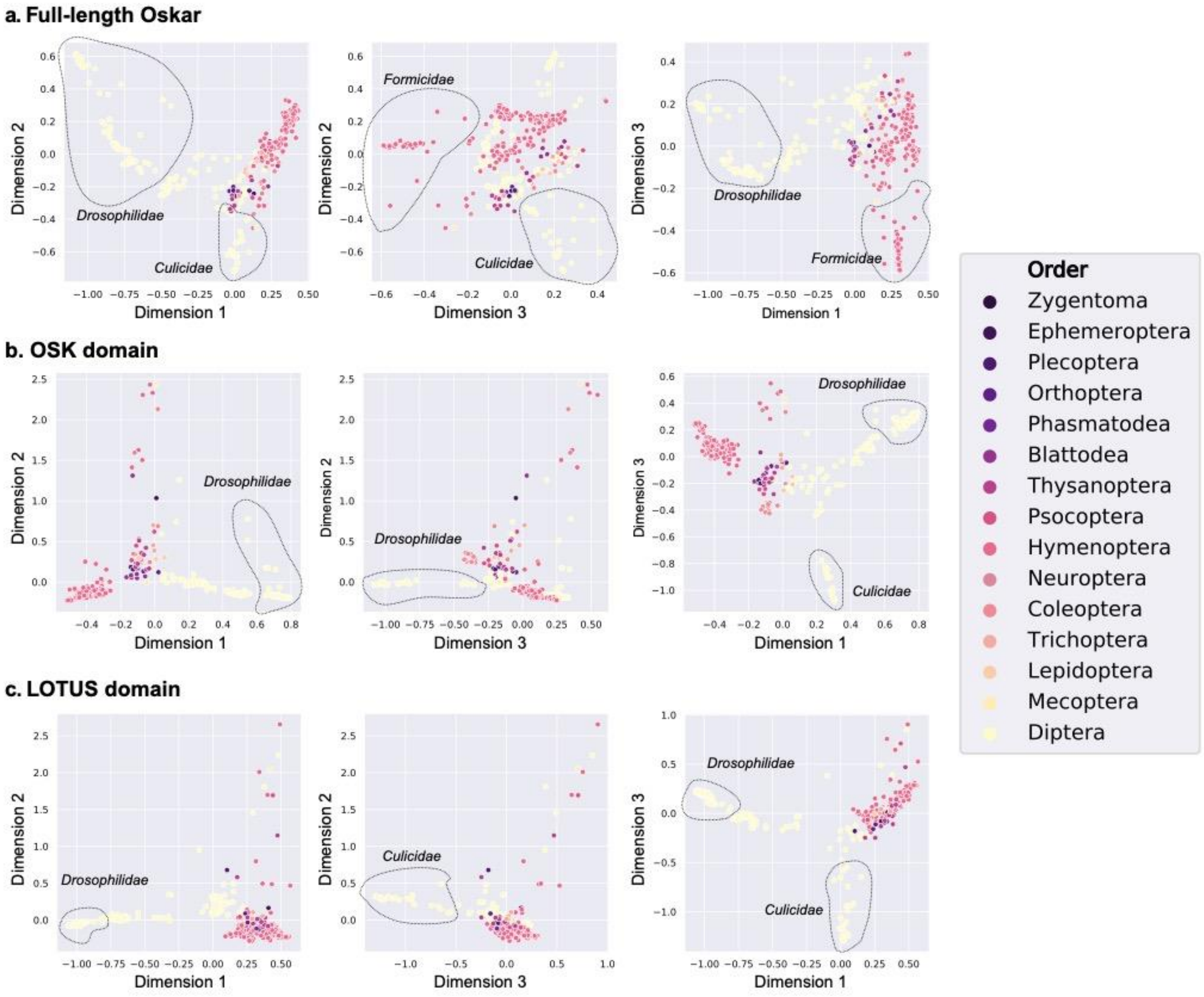

Supplementary Figure S11

a. LOTUS secondary structure conservation

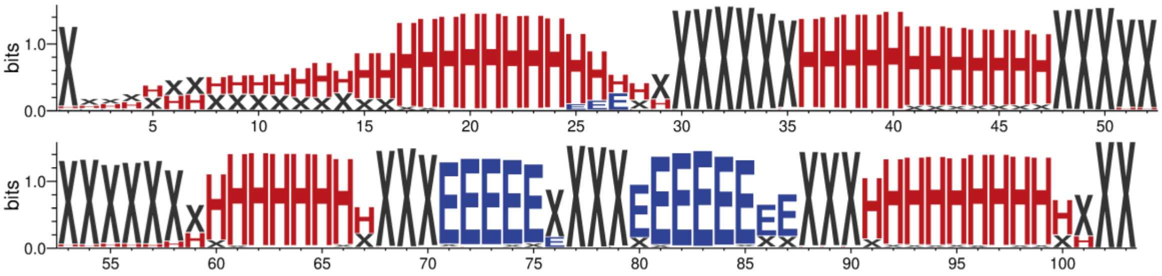

b. OSK secondary structure conservation

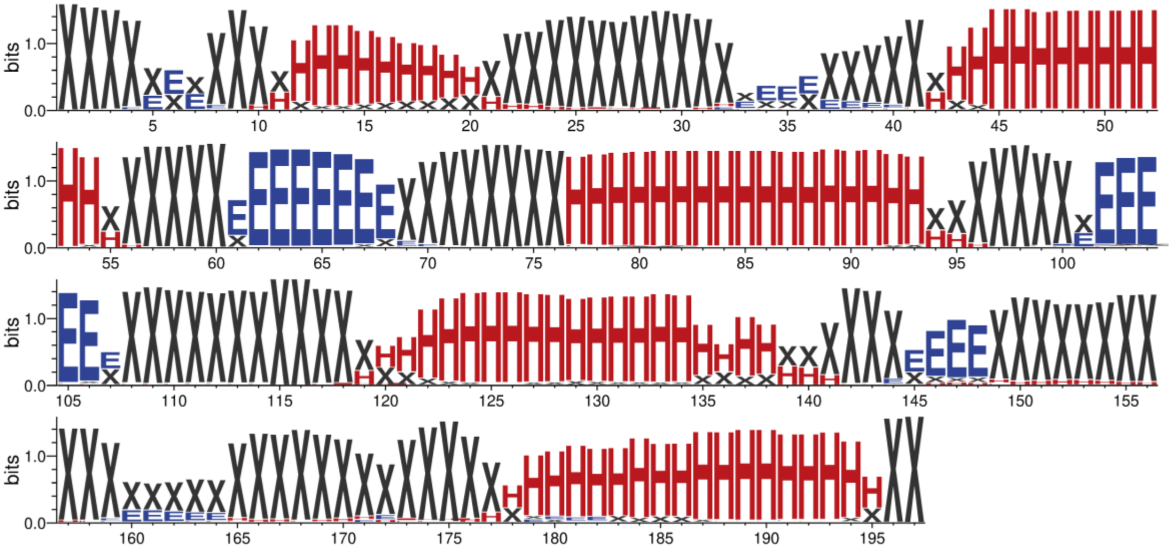

Supplementary Figure S12

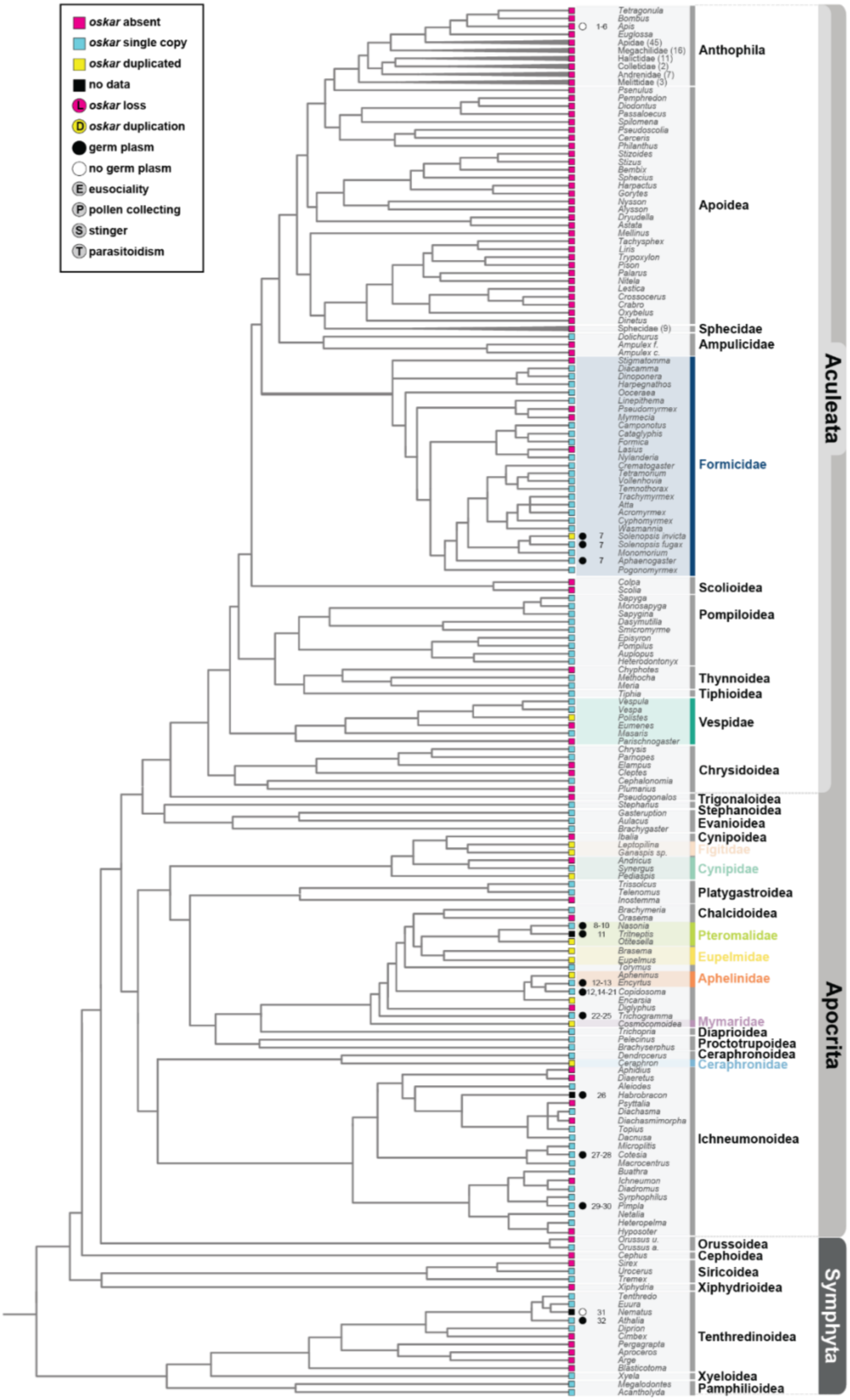

Supplementary Table Legends

Supplementary Table S1: Number of *oskar* sequences identified per order and per data source. Each row corresponds to an order and a data source: GCF: RefSeq; GCA: GenBank, TSA: Transcriptome Shotgun Assembly Database. “Filtered hits” column indicates the number of hits after the filtration algorithm described in the Methods is applied. Rightmost column defines the proportion of *oskar* sequences identified, as the number of datasets with a filtered hit divided by the total number of datasets searched.

Supplementary Table S2: Genome quality correlation to *oskar* identification. Mean and median values for the distributions of each indicated genome quality parameter, in which *oskar* was (a) or was not (b) identified. The means of both distributions are significantly different for all metrics (Mann Whitney U test, $p<0.05$). See Supplementary Figure S2 of graphical representation of distributions.

Supplementary Table S3. Assignment of metadata to germ line or brain categories. This table is found in ***Data>02_oskar_analyses/2/3/TableS3_germline_brain_table.csv*** at the GitHub repository https://github.com/extavourlab/Oskar_Evolution.

Supplementary Table S4. Models used to create protein sequence databases. This table shows which models were used to run the *ab initio* gene detection algorithm Augustus as described in Methods and Materials. Column order corresponds to any GCA dataset of an organism from this order. “Family” column is only used if a member of this order but of a different family was used. Finally, “augustus_model” shows which GCF dataset or premade augustus model, was used to run the gene prediction. This table is found in ***Data>Tables>TableS4_models.csv*** at the GitHub repository https://github.com/extavourlab/Oskar_Evolution.

Supplementary Table S5. *oskar* search results master table. This table summarizes all results of the *oskar* search performed on each dataset. Each row corresponds to a dataset. Columns are as follows: Id: the dataset NCBI identifier; Species: the organism’s species name; Family_name: the organism’s family name; Order_name: the organism’s order name; Hits: the number of sequences in the dataset found that satisfy our criteria for *oskar* orthology; Source: the NCBI database from which this dataset was downloaded; Filtered_hits: the number of *oskar* sequins in remaining the dataset after the filtration process was applied to all identified *oskar* sequences. For more information on the criteria used for *oskar* orthology and the filtration process, please see the Materials and Methods “*Identification of oskar orthologs*”. This table is found in ***Data>Tables>TableS5_models.csv*** at the GitHub repository https://github.com/extavourlab/Oskar_Evolution.

Supplementary Table S1

Insect Order	Source	Number of datasets searched	Total hits	Filtered hits	% of datasets with *oskar* identified
Archaeognatha	GCA	1	0	0	0
Archaeognatha	TSA	2	0	0	0
Blattodea	GCA	3	1	1	33.33
Blattodea	GCF	2	0	0	0
Blattodea	TSA	51	7	7	13.73
Coleoptera	GCA	12	1	1	8.33
Coleoptera	GCF	9	3	2	22.22
Coleoptera	TSA	86	31	14	16.28
Collembola	TSA	9	0	0	0
Dermaptera	TSA	7	0	0	0
Diptera	GCA	115	63	60	52.17
Diptera	GCF	43	58	43	100
Diptera	TSA	162	72	58	35.8
Embioptera	TSA	5	0	0	0
Ephemeroptera	GCA	2	0	0	0
Ephemeroptera	TSA	5	1	1	20
Grylloblattodea	TSA	2	0	0	0
Hemiptera	GCA	18	0	0	0
Hemiptera	GCF	12	0	0	0
Hemiptera	TSA	192	1	0	0
Hymenoptera	GCA	52	32	30	57.69
Hymenoptera	GCF	47	36	32	68.09
Hymenoptera	TSA	301	157	128	42.52
Lepidoptera	GCA	80	0	0	0
Lepidoptera	GCF	17	0	0	0

Lepidoptera	TSA	135	24	4	2.96
Mantodea	TSA	13	0	0	0
Mantophasmatodea	TSA	2	0	0	0
Mecoptera	TSA	4	2	2	50
Megaloptera	TSA	3	0	0	0
Neuroptera	TSA	7	1	1	14.29
Odonata	GCA	2	0	0	0
Odonata	TSA	7	0	0	0
Orthoptera	GCA	3	0	0	0
Orthoptera	TSA	28	2	2	7.14
Phasmatodea	GCA	13	0	0	0
Phasmatodea	TSA	31	6	2	6.45
Phthiraptera	GCF	1	0	0	0
Phthiraptera	TSA	7	0	0	0
Plecoptera	GCA	3	0	0	0
Plecoptera	TSA	8	3	3	37.5
Psocoptera	TSA	23	5	5	21.74
Raphidioptera	TSA	3	0	0	0
Siphonaptera	GCF	1	0	0	0
Siphonaptera	TSA	4	0	0	0
Strepsiptera	GCA	1	0	0	0
Strepsiptera	TSA	2	0	0	0
Thysanoptera	GCA	1	1	1	100
Thysanoptera	GCF	1	1	1	100
Thysanoptera	TSA	11	10	8	72.73
Trichoptera	GCA	3	1	1	33.33
Trichoptera	TSA	7	2	2	28.57
Zoraptera	TSA	2	0	0	0
Zygentoma	TSA	4	1	1	25

Crustacea	TSA	168	0	0	0
Crustacea	GCF	1	0	0	0
Crustacea	GCA	11	0	0	0

Supplementary Table S2

	Genome parameter	(a) *oskar* Identified	(b) *oskar* not identified	ratio (a):(b)
# contigs	mean	255,015	43,280	5.89
	median	69,255	20,653	3.35
# scaffolds	mean	182,706	23,596	7.74
	median	40,960	9,398	4.36
contig N50 (bp)	mean	324,036	726,696	0.45
	median	14,052	40,079	0.35
scaffold N50	mean	2,636,825	5,695,299	0.46
	median	96,730	385,460	0.25
contig L50	mean	40,955	3,701	11.07
	median	6,868	1,300	5.28
scaffold L50	mean	27,269	1,500	18.18
	median	1,131	191	59.53
# contigs per genome length	mean	0.00060	0.00017	3.53
	median	0.00021	0.00009	2.33
# scaffolds per genome length	mean	0.00045	0.00009	5.00
	median	0.00013	0.00005	2.60
